# Spice related catatonia and its treatment: The case study

**DOI:** 10.1192/j.eurpsy.2021.1543

**Published:** 2021-08-13

**Authors:** O. Pityk, O. Lutska, A. Sikora

**Affiliations:** 1 Psychiatry, Narcology And Medical Psychology Department, Ivano-Frankivsk National Medical University, Ivano-Frankivsk, Ukraine; 2 Department Of The First Psychotic Episode And Crisis Conditions, Precarpathian Regional Clinical Center of Ivano-Frankivsk Regional Council, Ivano-Frankivsk, Ukraine

**Keywords:** synthetic cannabinoids, oneiroid catatonia, management, Spice

## Abstract

**Introduction:**

Synthetic Cannabinoids were recognized as drugs of abuse since 2008, they are sold under various names (Kush, K2, Spice).

**Objectives:**

The Patient N., 17 years old male was admitted to the hospital with a first-time psychotic episode. He is fond of extreme sports (ski jumping, gymnastic and acrobatic elements).

**Methods:**

The patient was fully examined.

**Results:**

Then he was taken to the admission department. About a week before admission he fell, injuring his leg and head. After that he did not sleep, became excited, aggressive, “said strange things”. During the admission patient showed disorganized behavior, agitation, paranoid ideation, beliefs that others were inserting thoughts into his head (“thought insertion”) and that his thoughts could be read by others (“thought broadcasting”), imperative “voices”, bizarre delusional thoughts. The UDT showed positive K2 analysis. The patient was prescribed Diazepam, Zuclopenthixol, Valproic acid. No improvement observed. Haloperidol was prescribed next day. Then Haloperodol was changed to Quetiapine, and after 10 days of treatment no improvement was observed. The patient started to show catatonia symptoms such as elective mutism, mild rigidity, signs of cog-wheeling or clasp-knife rigidity. He experienced anxiety, fear, did not take care of himself. Every day he started to be aggressive, impulsive, started to experience auditory hallucinations. Due to that fact it was decided to prescribe Haloperidol, Chlorpromazine, Phenazepame, Diphenhydramine.That treatment improved behavior.

**Conclusions:**

Thus, the intensive treatment with antipsychotic medications in combination with benzodiazepines and diphenhydramine is much more preferable for the management of the cases of oneiroid catatonia due to the usage of Spice.

